# Distinct vitellogenin domains differentially regulate immunological outcomes in invertebrates

**DOI:** 10.1074/jbc.RA120.015686

**Published:** 2020-12-01

**Authors:** Weikang Sun, Hao Li, Yuehong Zhao, Longwei Bai, Yukai Qin, Qun Wang, Weiwei Li

**Affiliations:** Laboratory of Invertebrate Immunological Defense and Reproductive Biology, School of Life Sciences, East China Normal University, Shanghai, China

**Keywords:** invertebrate immunology, bacterial infection, vitellogenin (Vg), polymeric immunoglobulin receptor (pIgR), hemocyte, phagocytosis, CPZ, chlorpromazine, *Es*pIgR, *E. sinensis* polymeric immunoglobulin receptor, IPTG, isopropyl b-D-1-thiogalactopyranoside, LB, Luria–Bertani, LLTP, large lipid transfer protein, NJ, neighbor-joining, ORF, open reading frame, pIg, polyimmunoglobulin, pIgR, polymeric immunoglobulin receptor, SC, secretory component, TBS, Tris-buffered saline, Vg, vitellogenin, VWD, von Willebrand factor type D domain

## Abstract

The classical role of Vitellogenin (Vg) is providing energy reserves for developing embryos, but its roles appear to extend beyond this nutritional function, and its importance in host immune defense is garnering increasing research attention. However, Vg-regulated immunological functions are dependent on three different domains within different species and remain poorly understood. In the present study, we confirmed three conserved VG domains—LPD_N, DUF1943, and VWD—in the Chinese mitten crab (*Eriocheir sinensis*), highlighting functional similarities of Vg in vertebrates and invertebrates. Of these three domains, DUF1943 and VWD showed definitive bacterial binding activity *via* interaction with the signature components on microbial surfaces, but this activity was not exhibited by the LPD_N domain. Antibacterial assays indicated that only the VWD domain inhibits bacterial proliferation, and this function may be conserved between different species due to the conserved amino acid residues. To further explore the relationship between Vg and polymeric immunoglobulin receptor (pIgR), we expressed *Es*pIgR and the three *E. sinensis* Vg (*Es*Vg) domains in HEK293T cells, and coimmunoprecipitation assay demonstrated that only the DUF1943 domain interacts with *Es*pIgR. Subsequent experiments demonstrated that *Es*Vg regulates hemocyte phagocytosis by binding with *Es*pIgR through the DUF1943 domain, thus promoting bacterial clearance and protecting the host from bacterial infection. To the best of our knowledge, our work is the first to report distinct domains in Vg inducing different immunological outcomes in invertebrates, providing new evidence that pIgR acts as a phagocytic receptor for Vg.

Vitellogenin (Vg) is a member of the large lipid transfer protein (LLTP) superfamily, which also includes the microsomal triglyceride transfer protein and apolipoprotein (1, 2). As an egg yolk precursor protein, Vg is present in the females of nearly all oviparous species, including fish, amphibians, reptiles, birds, most invertebrates, and the platypus (3). Vg is usually synthesized extra-ovarianly and then transported by the circulation system to the ovaries, where it is internalized into growing oocytes *via* receptor-mediated endocytosis and then proteolytically cleaved to generate the yolk proteins lipovitellin (Lv) and phosvitin (Pv), providing nutrients to developing embryos (4). However, there is an increasing body of evidence indicating that its roles extend beyond this nutritional function, and its role in host immune defense is attracting considerable research attention (5).

For instance, recent studies have shown that Vg in vertebrate plays a role in its immune response, either as a pattern recognition molecule that recognizes bacteria or as an opsonin to enhance macrophage phagocytosis (6, 7). Furthermore, Vg has been reported to directly kill bacteria *via* interactions with lipopolysaccharides and lipoteichoic acid present in bacterial cell walls and to neutralize viruses by binding to and creating cross-links between virions (8). Several studies on the immunological functions of Vg in invertebrate have also been reported. For example, Vg in the mosquito *Anopheles gambiae* is able to interfere with antiplasmodium response by reducing the parasite-killing efficiency of the antiparasitic factor TEP1 (9). Vg in honeybees also has immunological binding properties and is able to mediate previous unexpected trans-generational immunity by transporting microbe-derived molecules into developing eggs (10, 11).

Vg is a high-molecular-mass glycolipophosphoprotein that typically circulates in the blood/hemolymph as a homodimer. It is encoded by multiple genes in several species including insects, fish, and frogs (12). Vg is considered to have similar characteristics in vertebrates, such as fish, and invertebrates, particularly crustacea (5). Typically, Vg contains three conserved domains: LPD_N (*i.e.*, vitellogenin N or LLT), which has been identified in the N-terminus of LLTP members; DUF1943, the function of which is unclear; and the von Willebrand factor type D domain (VWD), which is located at the C-terminus and distributed over a wide range of proteins (13). The N-terminal LPD_N domain encodes the Lv heavy chain (LvH), whereas the C-terminal VWD encodes the β-component (5).

Scanning electron microscopy as well as bacterial cell and protoplast lysis assay studies of fish have shown that Vg is able to lyse both *Escherichia coli* and *Staphylococcus aureus*, and the coating *E. coli*, *S. aureus*, and *P. pastoris* with Vg facilitates the phagocytosis of these microbes by macrophages (7), indicating that Vg is an opsonin, *i.e.*, a bridging molecule between microbes and macrophages. However, whether this function of Vg is conserved within invertebrates still remains to be demonstrated. Furthermore, the possible bacterial binding and phagocytosis-promoting functions mediated by the LPD_N, DUF1943, and VWD domains are still largely unknown, for both vertebrates and invertebrates, as is the phagocytic receptor that binds and regulates Vg-promoted bacterial phagocytosis.

## Results

### *Es*Vg binds to bacteria and exerts antibacterial activity *via* different domains

**Figure 1:**
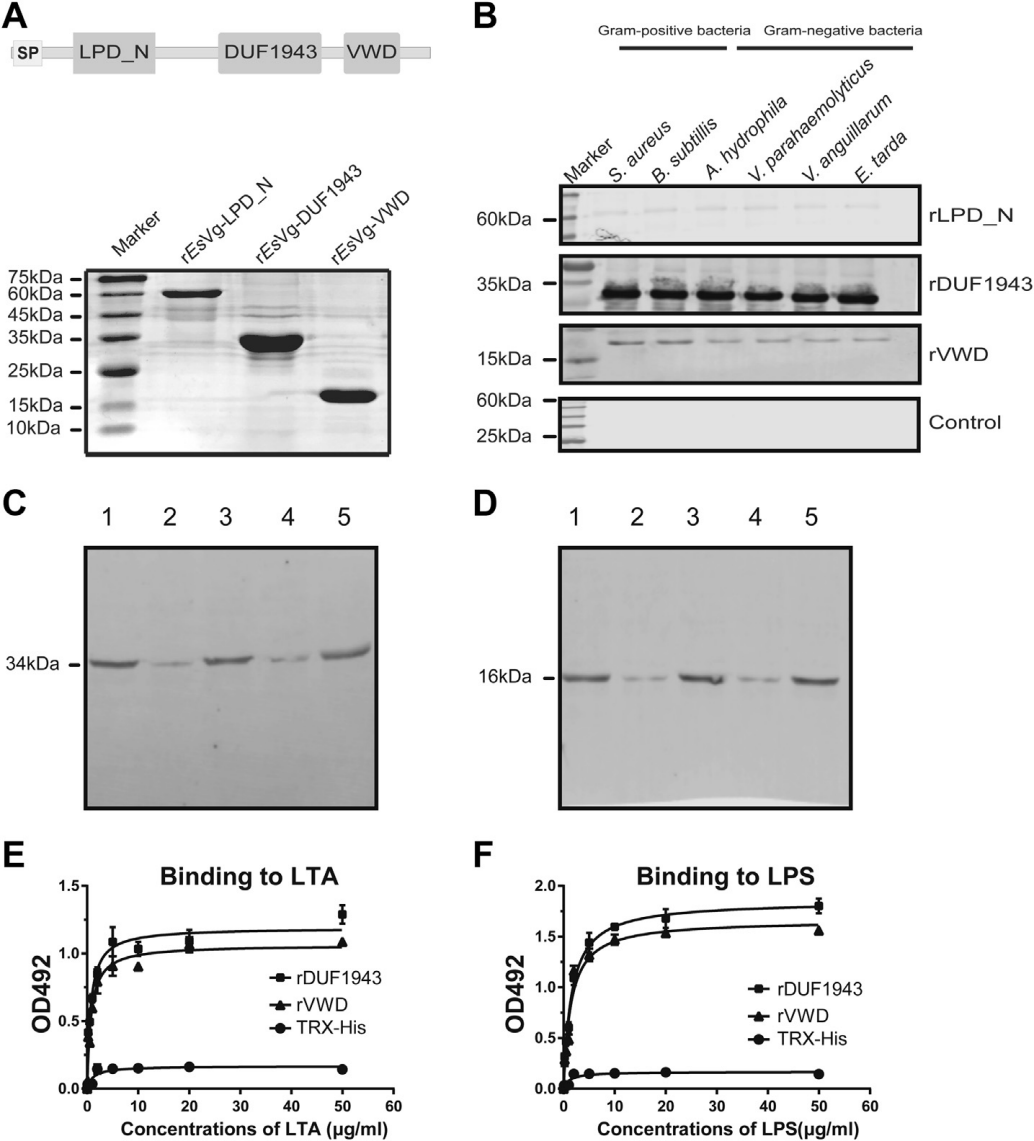
***Es*Vg binds to bacteria through different domains.***A*, expression of different r*Es*Vg domains in truncated proteins. Proteins were expressed in *E. coli* and then purified by affinity chromatography with Ni-NTA beads. *Top panel*, schematic illustration of the partial expression of *Es*Vg; *Bottom panel*, SDS-PAGE of the purified proteins. *B*, binding properties of *Es*Vg to bacteria. Western blotting was performed to analyze six bacterial strains incubated with r*Es*Vg-LPD_N, r*Es*Vg-DUF1943, and r*Es*Vg-VWD (rLPD_N, rDUF1943, rVWD). Following three washes with PBS, bacterial cell pellets and the supernatant from the final PBS wash (control) were analyzed. The bacterial strains are *Staphylococcus aureus*, *Bacillus subtilis*, *Aeromonas hydrophila*, *Vibrio Parahaemolyticus*, *Vibrio anguillarum*, and *Edwardsiella tarda*. *C*–*F*, binding of rDUF1943 and rVWD to LTA and LPS. Binding of rDUF1943 (*C*) and rVWD (*D*) to *S. aureus* and *V. Parahaemolyticus* was inhibited by the presence of LTA and LPS respectively. Lane 1, purified recombinant proteins; lane 2, *S. aureus* incubated with recombinant proteins, which were preincubated with LTA; lane 3, *S. aureus* incubated with recombinant proteins, which were preincubated with PBS; lane 4, *V. Parahaemolyticus* incubated with recombinant proteins, which were preincubated with LPS; lane 5, *V. Parahaemolyticus* incubated with recombinant proteins, which were preincubated with PBS. Quantitative binding of rDUF1943 and rVWD to LTA (*E*) and LPS (*F*) was analyzed by ELISA. TRX-His-tag peptide was used as control. Data are representative of at least three independent experiments.

Vg is widely considered to have the ability to bind bacteria (6, 7). However, the specific domains where Vg binds to bacteria are still very unclear in both vertebrates and invertebrates. To investigate whether *Es*Vg binds to different kinds of bacteria and to identify which domain performs the function of binding bacteria, recombinant proteins of different *Es*Vg domains, *i.e.*, r*Es*Vg-LPD_N, r*Es*Vg-DUF1943, and r*Es*Vg-VWD, were expressed in *E. coli* (Fig. 1*A*). The proteins were used in bacteria-binding assays to determine their ability to bind bacteria. Six different kinds of Gram-positive and Gram-negative bacteria were fully coated by r*Es*Vg. Interestingly, as shown in Figure 1*B*, the DUF1943 domain has a strong bacteria-binding ability for Gram-positive and Gram-negative bacteria. Furthermore, the VWD domain has a comparative weak bacteria-binding ability for Gram-positive and Gram-negative bacteria. However, the results clearly show that the LPD_N domain of *Es*Vg has no bacteria-binding ability.

To better understanding the microbial-binding activity of *Es*Vg, Western blotting and ELISA were employed to study the effects of LTA and LPS in Gram-positive and Gram-negative bacteria on the binding of rDUF1943 and rVWD to microbes. Results have shown that the binding of rDUF1943 and rVWD to *S. aureus* and *Vibrio parahaemolyticus* was inhibited by LTA and LPS, respectively (Fig. 1, *C*–*D*), which indicated that these domains bound to the Gram-positive and Gram-negative bacteria *via* interaction with the signature components on microbial surfaces. ELISA was performed to verify the possible interaction between rDUF1943 and rVWD with LTA and LPS. Results have shown that both rDUF1943 and rVWD protein had a significantly stronger (*p* < 0.05) affinity to the LTA (Fig. 1*E*) and LPS (Fig. 1*F*), which demonstrated that DUF1943 and VWD domain in *Es*Vg may specifically recognize Gram-positive and Gram-negative bacteria *via* LTA and LPS, respectively.

### Bacteria growth inhibition of *Es*Vg

**Figure 2:**
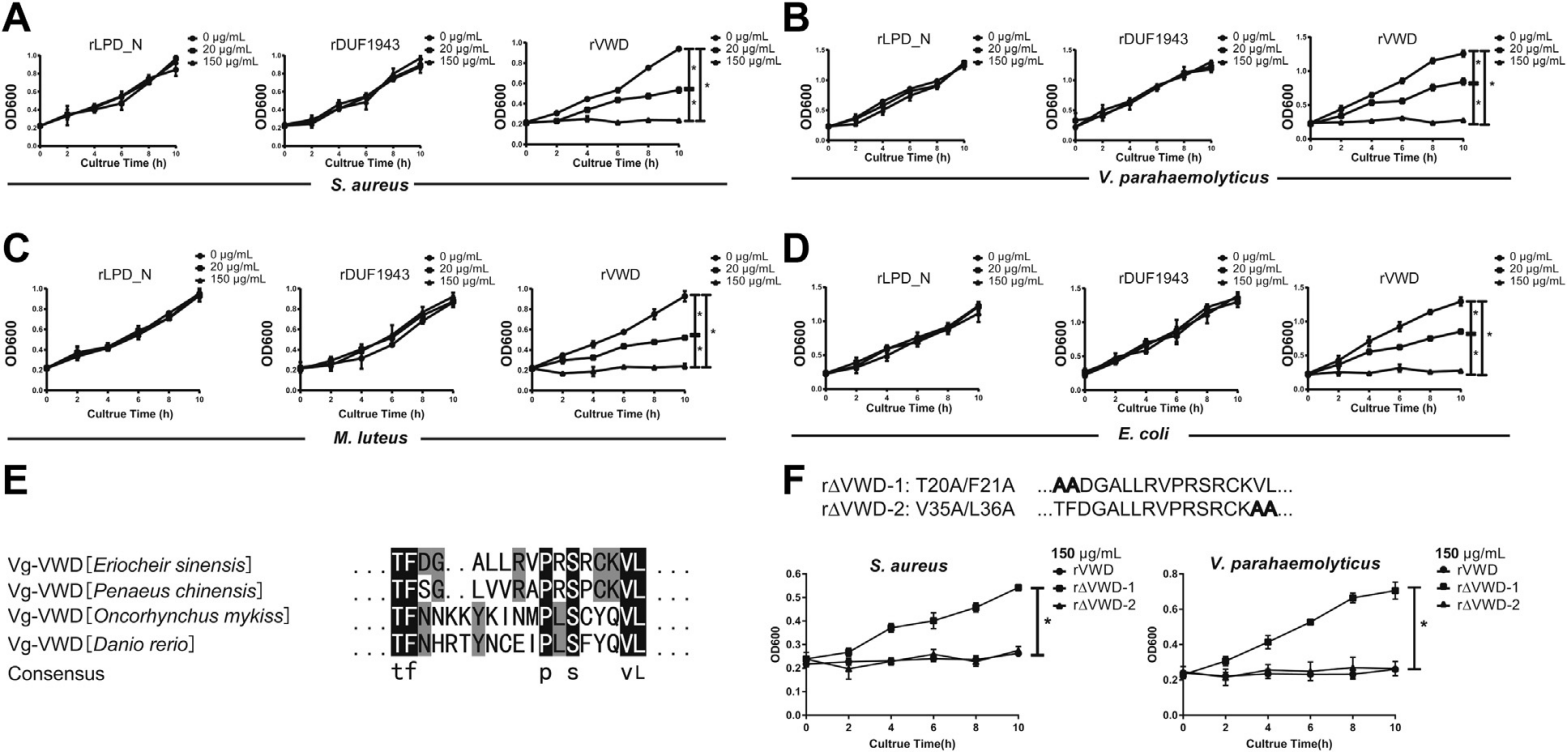
**Growth suppression effect of the varied domain of *Es*Vg against different bacteria.***A*–*D*, growth suppression effect of the LPD_N, DUF1943, and VWD domain of *Es*Vg against *S. aureus* (*A*), *V. parahaemolyticus* (*B*), *M. luteus* (*C*), and *E. coli* (*D*). Bacteria were washed and suspended in the same volume of TBS with 0 μg/ml, 20 μg/ml or 150 μg/ml *Es*Vg. *E*, sequence alignment between VWD from four species showed comparative similar regions from *E. sinensis* T20 to L36. *F*. mutated *E. sinensis* rVWD with indicated point mutants that displayed in bold and named r△VWD-1 and r△VWD-2, respectively (upper panel), growth suppression effect of the r△VWD-1 and r△VWD-2 of *Es*Vg against *S. aureus* and *V. parahaemolyticus* (lower panel). The OD600 was measured every 2 h to obtain a growth curve of the different samples. TBS was used as the control. Data are means ± SD of triplicate experiments. Statistical significance was determined by one-way analysis of variance and post-hoc Duncan's multiple range tests. ∗*p* < 0.05.

Considering the possible role of *Es*Vg in innate immune defense and binding bacteria (14, 15, 16, 17), we investigated whether these domains of *Es*Vg inhibited the growth of bacteria. The inhibitory effects of the rLPD_N, rDUF1943, and rVWD of *Es*Vg on the growth of *S. aureus* (Fig. 2*A*), *Vibrio parahaemolyticus* (Fig. 2*B*), *M. luteus* (Fig. 2*C*), and *E. coli* (Fig. 2*D*) were analyzed. Results have shown that bacteria growth was not inhibited by 0 mg/ml of each recombinant protein, and only rVWD could significantly inhibit the growth of different bacteria in a dose-dependent manner, rather than rLPD_N and rDUF1943. Collective results suggest the unique role of VWD domain in bacteria growth inhibition while the LPD_N and DUF1943 domains may participate in other immune responses. To understanding the function of conserved amino acid residues among VWD domain from different species on bacterial growth inhibition, four VWD sequences from vertebrate and invertebrate were aligned, and results have shown comparable similar regions from *E. sinensis* T20 to L36 (Fig. 2*E*). After mutating the T20/F21 (r△VWD-1) and V35/L36 (r△VWD-2) residues, the results have shown that r△VWD-2 cannot affect rVWD inhibited bacterial growth, while r△VWD-1 significantly affects rVWD inhibited bacterial growth (Fig. 2*F*), suggesting that T20/F21 amino acid residues that conserved between different species may have critical role on VWD inhibited bacterial growth.

### *Es*Vg enhances the phagocytosis of bacteria in hemocytes

*Es*Vg-regulated phagocytosis has been confirmed in several other species, and the domains of *Es*Vg have different bacteria-binding abilities (18, 19). Therefore, we investigated the ability of *Es*Vg to reduce the number of bacteria *via* phagocytosis regulation. For this purpose, we precoated different strains of FITC-labeled bacteria with r*Es*Vg-DUF1943 and r*Es*Vg-VWD, which have shown bacterial binding activity.

**Figure 3:**
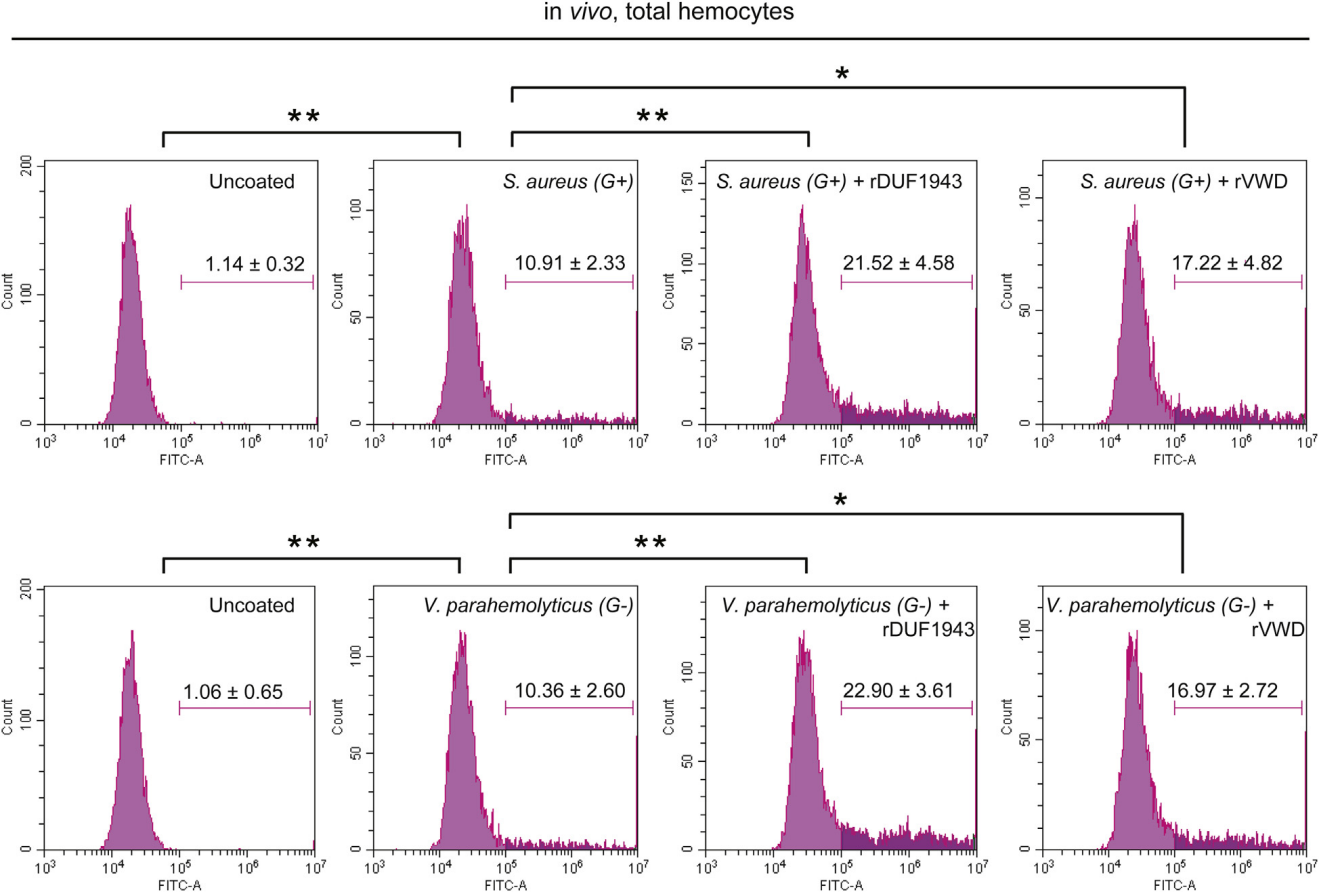
**Promotion of hemocytic phagocytosis by *Es*Vg.** Different domains of *Es*Vg promote phagocytosis of bacteria *in vivo*. *S. aureus* (*Top panel*) and *V. parahaemolyticus* (*Bottom panel*) were heat-inactivated and labeled with FITC before coating with rDUF1943 and rVWD, respectively. The bacteria were then injected into the hemolymph and hemocytes were collected 1 h later for flow cytometric analysis. A total of 10,000 hemocytes were counted for each sample. Three independent repeats were performed, and results are expressed as the mean ± SD. ∗*p* < 0.05, ∗∗*p* < 0.01 (Student’s *t* test).

*In vivo* phagocytosis assays demonstrated that r*Es*Vg-DUF1943 strongly enhances the rate of *S. aureus* and *V. parahaemolyticus* phagocytosis by approximately 100% (Fig. 3), and r*Es*Vg-VWD improves the rate of *S. aureus* and *V. parahaemolyticus* phagocytosis by approximately 70% (Fig. 3). Collective results indicated that bacterial interacted *Es*Vg may enhance hemocytes phagocytosis *via* a phagocytic receptor, which remain largely unexplored in both vertebrate and invertebrate.

### cDNA cloning and bioinformatics analysis of *Es*pIgR

The polymeric immunoglobulin receptor (pIgR) is an important component of the mucosal immune system. It mediates the transcytosis of polyimmunoglobulin (pIg) through epithelial cells to the mucosal surface to form SIgs, exerting immune defense functions in vertebrates (20, 21). However, information on pIgR in hemocyte endocytic processes remains limited. The possible relationship between Vg and pIgR has been reported in sea bass. Therefore, research on *E. sinensis* polymeric immunoglobulin receptor (*Es*pIgR) is particularly important.

**Figure 4:**
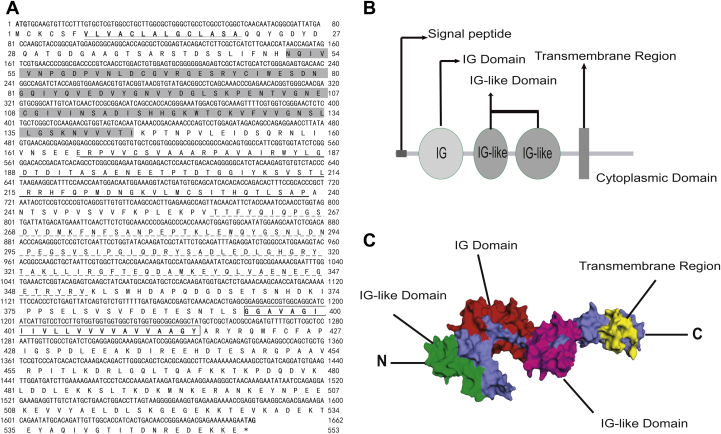
**Sequence information of *Es*pIgR.***A*, cDNA and amino acid sequences of *Es*pIgR. The sequence was subjected to on-line SMART analysis. The signal peptide is *double underlined*; the IG domain is *shaded*, and two IG-like domains are *single and dotted underlined*, respectively; the transmembrane segment is *boxed*. *B*, domain analysis of the putative *Es*pIgR protein by the SMART tool showing that it contains a signal peptide, a transmembrane segment, an IG domain, and two IG-like domains. *C*, a three-dimensional model of *Es*pIgR built by the online software SWISS-MODEL.

**Figure 5:**
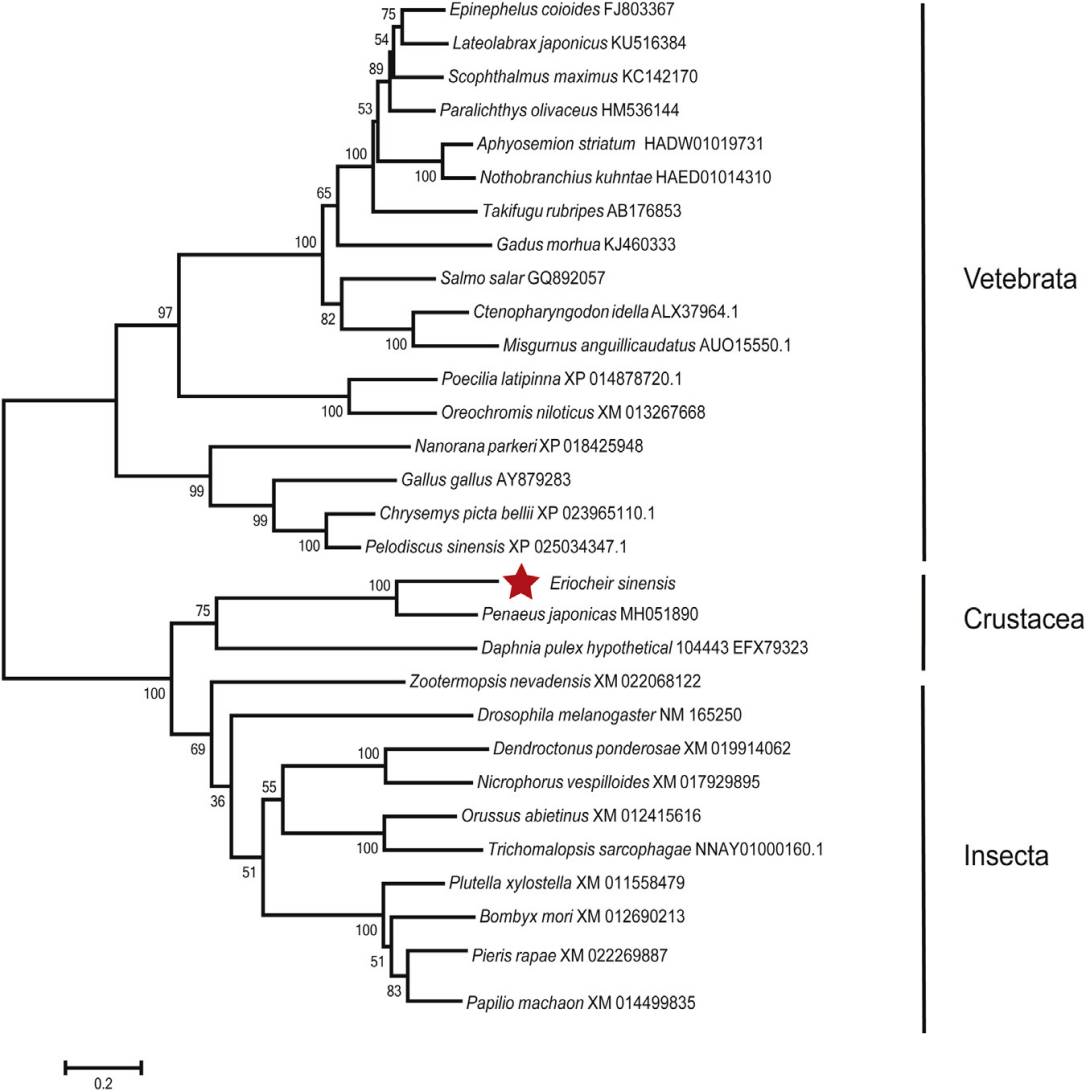
**Neighbor-joining (NJ) phylogenetic analysis of *Es*pIgR proteins.** Phylogenetic analysis of *Es*pIgR and some representative proteins from vertebrate and invertebrate species. The NJ phylogenetic tree was built in MEGA version 6 (*n* = 1000 bootstraps).

The 1662-bp open reading frame (ORF) region of *Es*pIgR was cloned from the hemocytes of *E. sinensis*. The putative *Es*pIgR protein contained 553 amino acids and an isoelectric point of 4.82, the ORF was predicted to encode a 60.7-kDa protein, and a signal peptide was located at their N terminus (Fig. 4*A*). Furthermore, three functional domains: an IG domain, two IG-like domains, and one transmembrane region were identified in the protein (Fig. 4, *B*–*C*). Phylogenetic analysis revealed three major branches that included vertebrates, crustacea, and insecta, with *Es*pIgR clustered within the crustacea and close to insects (Fig. 5).

### Expression pattern of *Es*pIgR

**Figure 6:**
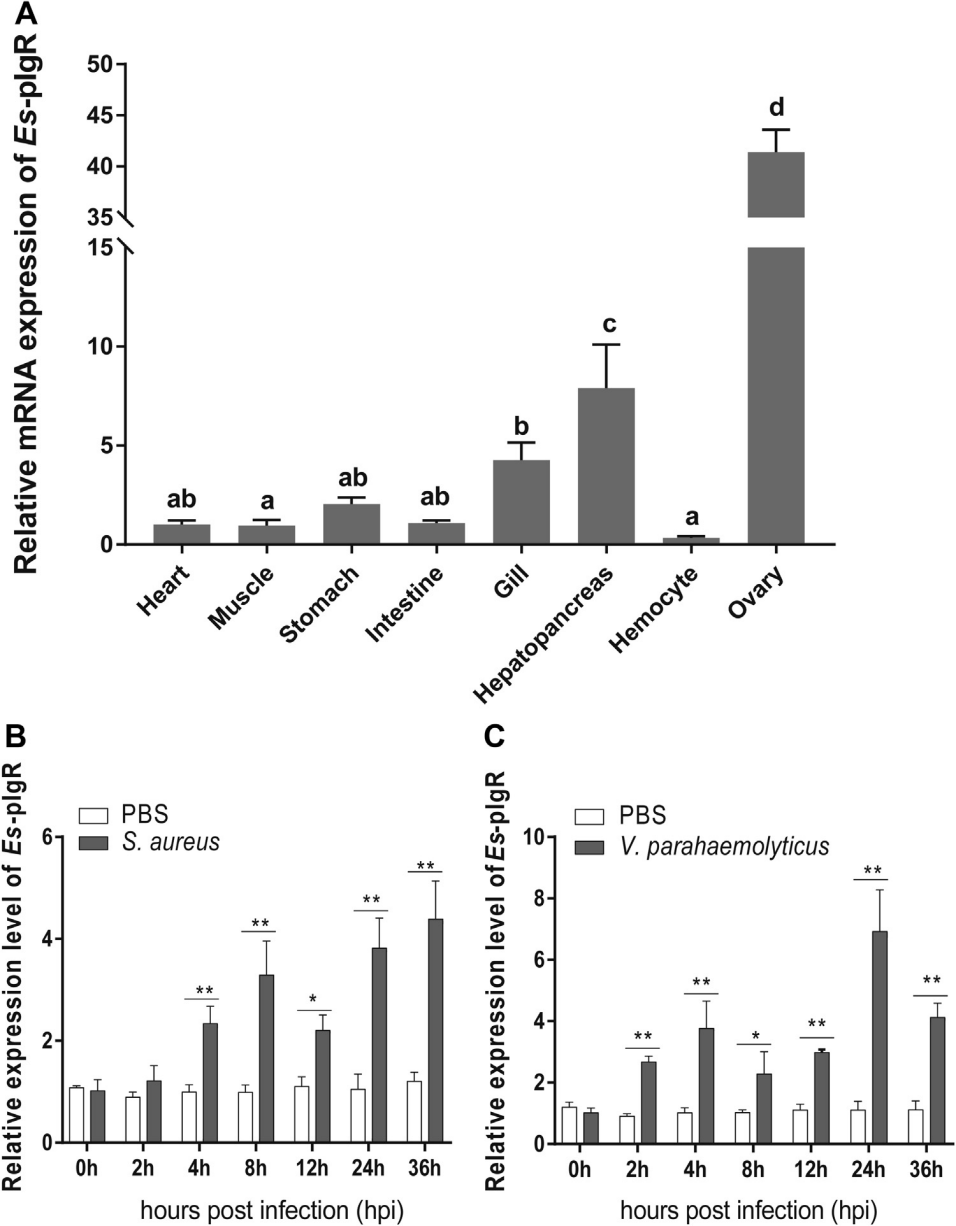
**Expression pattern of *Es*pIgR.***A*, tissue distribution of *Es*pIgR. RNA samples were extracted from healthy *E. sinensis* crabs, and *Es*pIgR expression was studied by RT-PCR (*β*-actin was the internal reference). Each sample was taken from at least three crabs, shown are the means ± SD. Three independent repeats were performed (≥5 crabs per sample), with different lowercase indicated the significance (one-way ANOVA). *B*–*C*, expression profiles of *Es*pIgR mRNA in crab hemocytes after infection by *S. aureus* (*B*) and *V. parahaemolyticus* (*C*). RNA was extracted at each time point. qRT-PCR was used to check the expression of *Es*pIgR in each sample, with *β*-actin as the reference. Shown are the means ± SD. Three independent repeats were performed (≥5 crabs per sample). ∗*p* < 0.05, ∗∗*p* < 0.01 (Student’s *t*-test).

The *Es*pIgR gene is expressed in different tissues harboring hemocytes (Fig. 6*A*), which indicates its role in crab immune regulation. To test whether *Es*pIgR expression could be induced postinfection, *S. aureus* (Fig. 6*B*) and *V. parahaemolyticus* (Fig. 6*C*) were used to infect crabs *in vivo*. Soon after infection with either pathogen, *Es*pIgR expression is significantly induced, thus demonstrating its potential participation in innate immunity.

### *Es*Vg binding with *Es*pIgR

**Figure 7:**
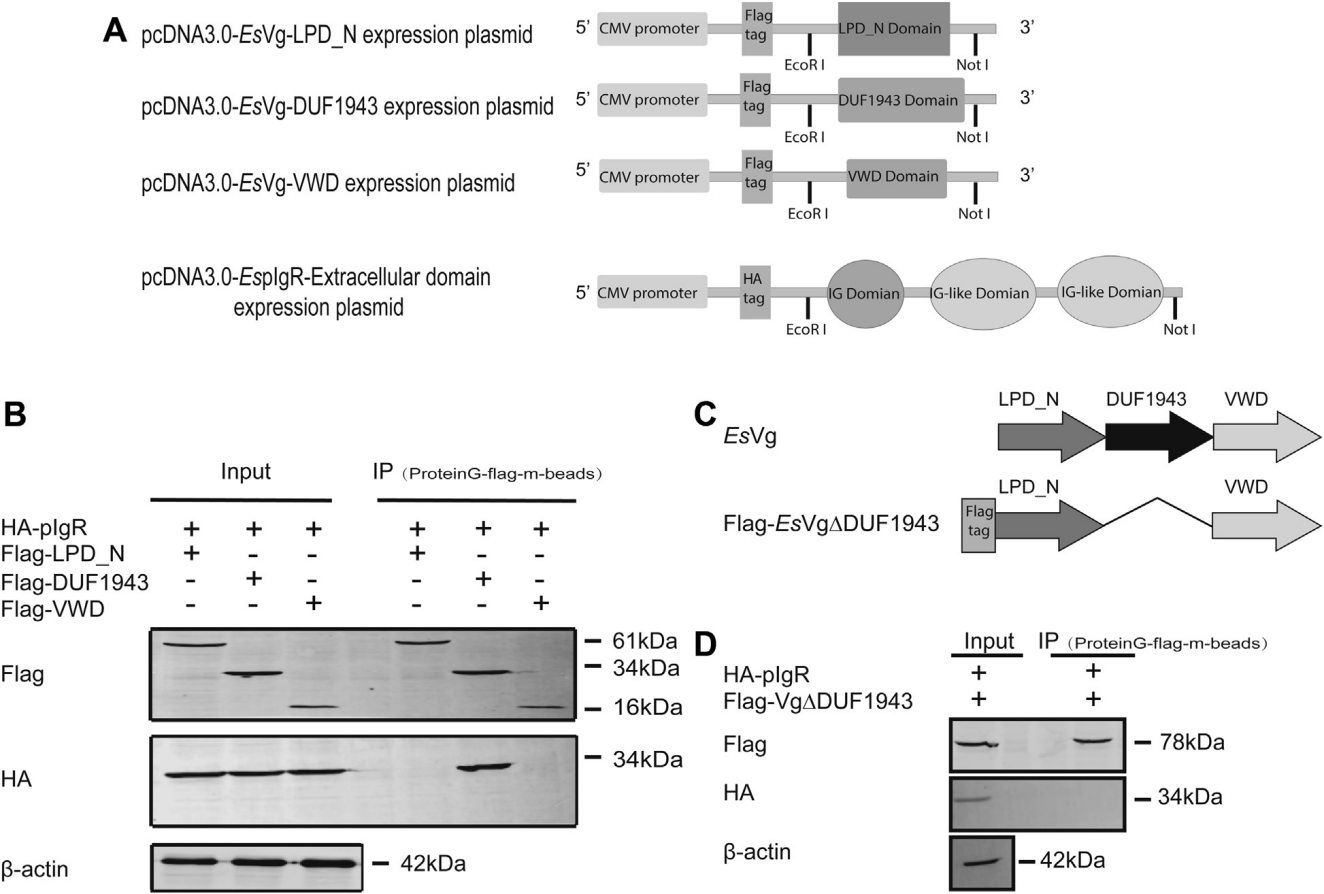
***Es*Vg binding with *Es*pIgR *via* DUF1943 domain.***A*, illustration of *Es*pIgR and *Es*Vg expression plasmids. *B*, coimmunoprecipitation assay results showing the interaction between different domains of *Es*Vg proteins and *Es*pIgR in HEK293T cells. Anti-FLAG and anti-HA antibodies were used to detect *Es*Vg and *Es*pIgR in coinfected HEK293T cells, respectively. The anti-HA and anti-FLAG antibodies were incubated with the cell lysates and then isolated using Protein G-FLAG-m-beads. *C*, schematic diagram of Flag labeled truncated *Es*Vg plasmid that without DUF1943 domain named Flag-*Es*Vg△DUF1943. *D*, coimmunoprecipitation assay results showing undetected interaction between Flag-*Es*Vg△DUF1943 and *Es*pIgR in HEK293T cells. Anti-FLAG and anti-HA antibodies were used to detect *Es*Vg and *Es*pIgR in coinfected HEK293T cells, respectively. Data are representative of at least three independent experiments.

*Es*pIgR may act as a potential receptor of *Es*Vg on the hemocyte membrane. To elucidate whether binding might occur between *Es*Vg and *Es*pIgR and which domains of *Es*Vg interact with *Es*pIgR, the extracellular domain of *Es*pIgR and three different domains of *Es*Vg cDNA plasmids (LPD_N, DUF1943, VWD, and △DUF1943) were constructed and transfected into HEK293T cells (Fig. 7*A*). The coimmunoprecipitation results demonstrated that each plasmid was well expressed in these cells. Specifically, the *Es*Vg-DUF1943 domain (FLAG-DUF) strongly binds with the *Es*pIgR protein (HA-*Es*pIgR). However, the *Es*Vg-LPD_N domain (FLAG-LPD) and the *Es*Vg-VWD domain (FLAG-VWD) did not bind with the *Es*pIgR protein (Fig. 7*B*). Moreover, expressed plasmid that only contained LPD_N and VWD (Fig. 7*C*) cannot interaction with pIgR (Fig. 7*D*), which confirmed the essential role of DUF1943 in protein interaction. Thus, the *Es*Vg-DUF1943 domain binds to the extracellular domain of *Es*pIgR protein whereas the *Es*Vg-LPD_N and *Es*Vg-VWD domains have no binding function with *Es*pIgR.

### *Es*Vg bind to bacteria and allow endocytosis through *Es*pIgR

**Figure 8:**
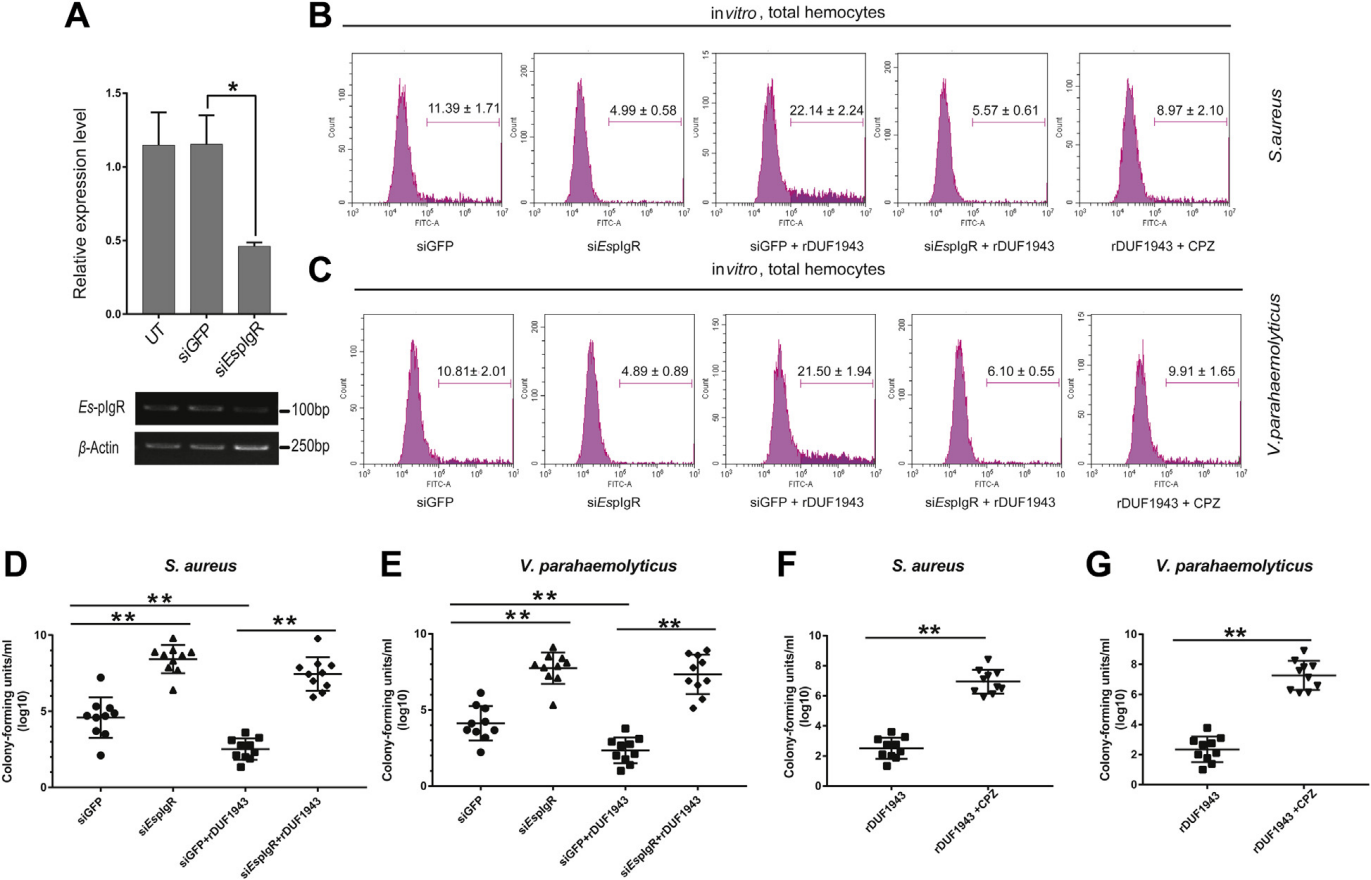
**Bacteria enters hemocytes *via Es*Vg-*Es*pIgR-clathrin-mediated endocytosis.***A*, effects of RNAi on *Es*pIgR. *Es*pIgR siRNA (si*Es*pIgR) was designed to knockdown *Es*pIgR expression, and its expression in crab hemocytes was determined 24 h postinjection with si*Es*pIgR by qRT-PCR (siGFP = control). Shown are means ± SD from three independent repeats (≥3 crabs per sample). ∗*p* < 0.05 (Student’s *t*-test). *B*–*C*, opsonin effect of the *Es*Vg-*Es*pIgR axis *in vitro*. *S. aureus* (*B*) and *V. parahaemolyticus* (*C*) were heat-killed, labeled with FITC, and fully coated with rDUF1943. The bacteria (1 × 10^6^ CFU) were incubated with 1 × 10^6^ hemocytes at 25 °C for 40 min. The mixture was centrifuged at 500*g* for 8 min to obtain the hemocytes. After washing, the hemocytes were analyzed by flow cytometry, where 10,000 hemocytes were counted for each sample. CPZ, an inhibitor of clathrin-mediated endocytosis, as well as rDUF1943-coated *S. aureus* (*B*) and *V. parahaemolyticus* (*C*) (1 × 10^8^ CFU) were injected. Hemocytes were collected 40 min later and subjected to flow cytometry analysis. The phagocytosis ratios were calculated from three independent experiments, and the graphs are representative of the three repeats. *D*–*E*, RNAi of *Es*pIgR or being fully coated by rDUF1943 affects bacterial proliferation in crab hemocyte medium supernatant. From each hemocytes medium, the supernatant was drawn at day 3 post-*S. aureus* (*D*) and post-*V. parahaemolyticus* (*E*) infection and plated onto agar plates for bacterial counting. *F*–*G*, injection of CPZ increases bacterial proliferation in crab hemocyte medium supernatant. The medium supernatant was drawn at day 3 post-*S. aureus* (*F*) and post-*V. parahaemolyticus* (*G*) infection and plated onto agar plates for bacterial counting.

The critical role of pIgR on phagocytosis regulation in crustacea has been reported (22), and it is implicated that Vg may interact with pIgR (23). Thus, to establish whether *Es*pIgR-*Es*Vg participates in the immune response against different bacteria by endocytosis, flow cytometry was performed to analyze the occurrence of hemocytic phagocytosis. siRNA was injected into crabs *in vitro*, and the expression of *Es*pIgR in hemocytes showed effective inhibition (Fig. 8*A*). Because the *Es*Vg-DUF1943 domain binds with both bacteria and *Es*pIgR, we precoated different strains of FITC-labeled bacteria with r*Es*Vg-DUF1943 and then performed phagocytosis assays *in vitro*. As shown in Figure 8*B*, after *S. aureus* stimulation, *Es*pIgR-silenced hemocytes show a lower percentage of hemocyte phagocytosis (∼4.99%) compared with that of the controls (∼11.39%). Moreover, r*Es*Vg-DUF1943 coating leads to a higher percentage of hemocyte phagocytosis (∼22.14%) compared with the controls (∼11.39%) *in vitro*. However, this phagocytosis process is inhibited by injecting si*Es*pIgR (∼5.57%). Similar results were also observed for the *V. parahaemolyticus* stimulation group (Fig. 8*C*), where the *in vitro* test showed that injection of si*Es*pIgR also inhibits phagocytosis, with the percentage of phagocytosis being ∼4.89% in the *Es*pIgR knockdown group. Conversely, r*Es*Vg-DUF1943 enhances the rate of *V. parahaemolyticus* phagocytosis (∼21.50%), and this enhancement is blocked after injection of si*Es*pIgR (∼6.10%). Thus, these results demonstrate the essential role of bacteria-specific binding with EsVg-EspIgR in phagocytosis.

To further confirm the role of the *Es*Vg-*Es*pIgR axis in the phagocytosis of bacteria by hemocytes *in vitro*, bacteria colonization levels in the hemocyte culture supernatant were studied. When *S. aureus* (Fig. 8*D*) or *V. parahaemolyticus* (Fig. 8*E*) was added together with si*Es*pIgR, the number of colonized bacteria in the hemocyte culture supernatant is significantly higher than in the controls. In addition, the fully coated r*Es*Vg-DUF1943 group exhibits reduced bacterial concentrations after stimulation with *S. aureus* (Fig. 8*D*) or *V. parahaemolyticus* (Fig. 8*E*). Furthermore, after RNAi of *Es*pIgR, the culture supernatant does not exhibit a colonization-inhibiting effect (Fig. 8, *D*–*E*). Thus, these results demonstrate that *Es*Vg interacts with *Es*pIgR on the hemocyte membrane and promotes innate immune defense *via* endocytosis after bacterial infection.

### *Es*Vg-*Es*pIgR axis mediates clathrin-dependent bacteria endocytosis

To investigate whether the *Es*Vg-*Es*pIgR-mediated endocytosis is clathrin-dependent, chlorpromazine (CPZ), an inhibitor of clathrin-mediated endocytosis (22), as well as r*Es*Vg-DUF1943-coated *S. aureus* and *V. parahaemolyticus*, was added to crab hemocytes culture. Then, flow cytometry was performed to investigate the occurrence of hemocytic phagocytosis. The results show that, after stimulated with *S. aureus*, CPZ causes a dramatic decrease in hemocytic phagocytosis (Fig. 8*B*). The percentage of phagocytosis is ∼22.14% in the r*Es*Vg-DUF1943 group, which is higher than that in the CPZ and r*Es*Vg-DUF1943 coinjection group (∼8.97%). Similar results are also observed for the *V. parahaemolyticus* injection group (Fig. 8*C*). The percentage of phagocytosis is ∼21.50% in the r*Es*Vg-DUF1943 group, which is higher than that in the CPZ and r*Es*Vg-DUF1943 coinjection group (∼9.91%). Thus, these data indicate that the ability of *Es*Vg to promote endocytosis is blocked by CPZ.

To confirm that the endocytosis of bacteria *via* the *Es*Vg-EspIgR axis is clathrin-dependent, bacterial concentration in the primary-cultured crab hemocytes after being stimulated with *S. aureus* (Fig. 8*F*) and *V. parahaemolyticus* (Fig. 8*G*) was detected. The concentration of bacteria in the CPZ-added group is significantly higher than that in the controls. Thus, the results indicate that *Es*Vg-*Es*pIgR-mediated endocytosis is clathrin-dependent.

### *Es*Vg-*Es*pIgR-mediated endocytosis protects the host from bacterial infection

**Figure 9:**
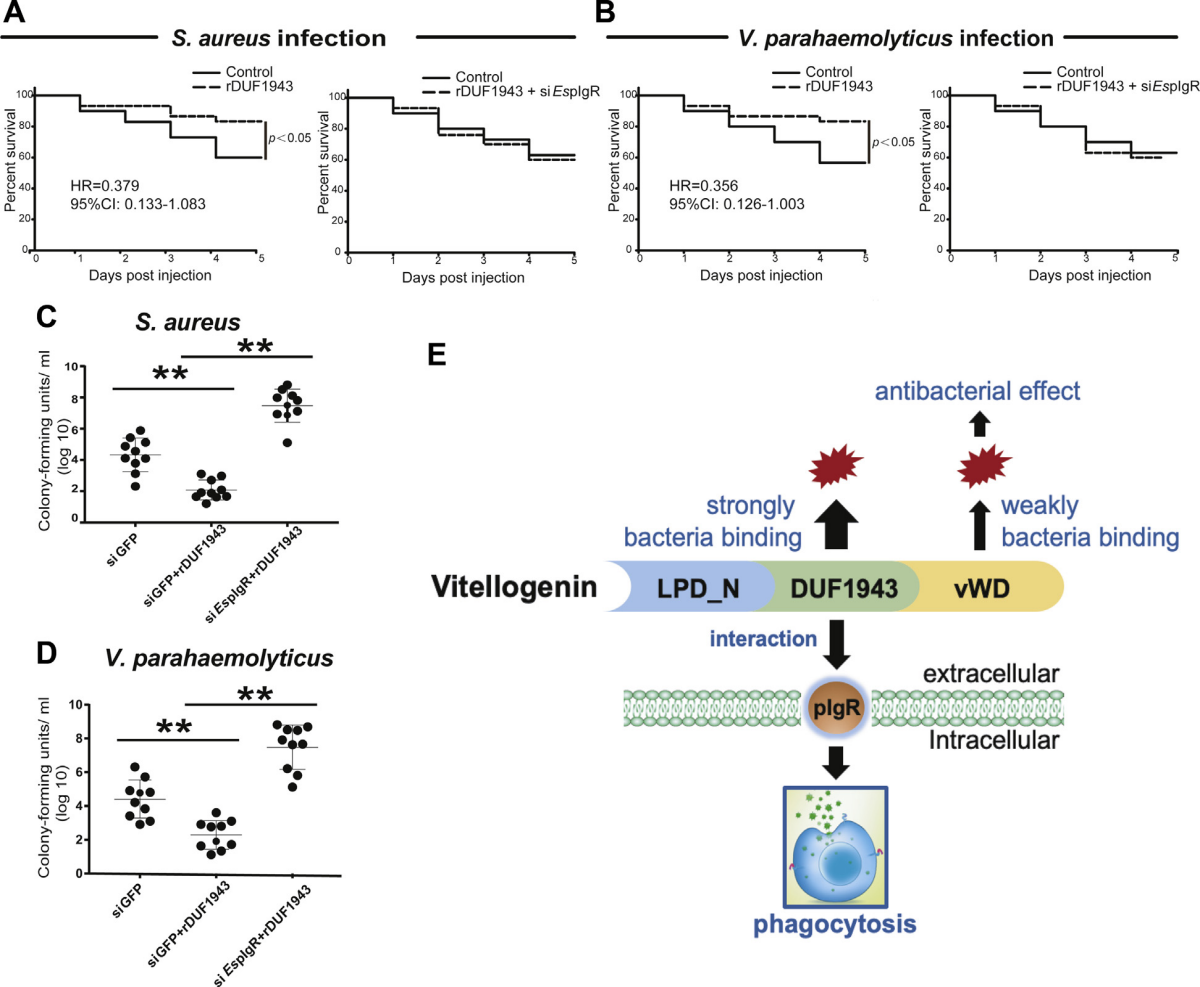
**Protection of the crab host from bacterial infection *via Es*Vg-*Es*pIg-mediated endocytosis.***A*–*B*, *Es*Vg recombinant proteins improve crab survival while knockdown of *Es*pIgR leads to crab death. Crabs were each injected with rDUF1943 and their 5-day survival post-*S. aureus* (*A*) and post-*V. parahaemolyticus* (*B*) infection was recorded from three independent repeats of 30 crabs per sample. In addition, survival rates with *S. aureus* (*A*) and *V. parahaemolyticus* (*B*) infection after being injected with si*Es*pIgR and rDUF1943 were also recorded. Univariate Cox proportional hazards regression models were performed to estimate the crude hazard ratios (HRs) or adjusted HRs and their 95% confidential intervals (CIs). SPSS version 11.5 (SPSS Inc, Chicago, IL) software was used for analyses. *C*–*D*, recombinant protein of *Es*Vg-DUF1943 reduces bacterial proliferation while RNAi of *Es*pIgR increases bacterial proliferation in crabs treated as described above. From each crab, the hemolymph was drawn at day 3 post-*S. aureus* (*C*) and post-*V. parahaemolyticus* (*D*) infection and plated onto agar plates for bacterial counting. *E*, schematic of the *Es*Vg-*Es*pIgR axis promoting bacteria endocytosis. Shown are the means ± SD. Three independent repeats were performed (≥5 crabs per sample). ∗∗*p* < 0.01 (Student’s *t*-test).

To verify the role of *Es*Vg-*Es*pIgR-mediated endocytosis in innate immunity, two kinds of bacteria were coated with r*Es*Vg-DUF1943 in the presence or absence of si*Es*pIgR and injected into crabs *in vivo*. Crab survival assay shown that r*Es*Vg-DUF1943 significantly improved percent survival rate of crab post both *S. aureus* (Fig. 9*A*) and *V. parahaemolyticus* (Fig. 9*B*) infection in *Es*pIgR-dependent manner. In coincidence, r*Es*Vg-DUF1943 significantly suppressed hemocytes bacterial concentration in *Es*pIgR-dependent manner post both *S. aureus* (Fig. 9*C*) and *V. parahaemolyticus* (Fig. 9*D*) infection. These results suggest that the *Es*Vg-*Es*pIgR axis plays critical roles in innate immunity by promoting hemocytic phagocytosis.

## Discussion

Vg was initially regarded as a female-specific protein (24). However, Vg synthesis, albeit in small quantities, has been shown to occur in males and sexually immature animals, indicating that the function of Vg extends beyond that as an energy reserve for the nourishment of developing embryos (25). Previous studies on fish and mosquitos have demonstrated the crucial role of Vg on bacterial binding (6, 7), bacterial killing (19), and phagocytosis regulation profiles (6), but how Vg protein contributes to such diverse conserved immunological roles remains unknown. In the present study, we revealed that crab Vg binds bacteria *via* the DUF1943 and VWD domains and exerts antibacterial effects in a VWD-domain-dependent manner. Moreover, *Es*Vg binds with *Es*pIgR using the DUF1943 domain and promotes bacterial phagocytosis in hemocytes to control antibacterial functions. To the best of our knowledge, this study provides the first evidence for the critical role of different domains in *Es*Vg in diverse antibacterial processes.

Vgs identified so far have been shown to share a similar domain structure combination. In most cases, Vgs include three conserved domains, *i.e.*, the LPD_N, DUF1943, and VWD domains (5). However, the functions of these domains in different species remain controversial. For instance, a study on zebrafish revealed that the DUF1943 and VWD domains constitute a pattern-recognition system capable of interacting with bacteria and that DUF1943 functions as an opsonin capable of promoting the phagocytosis of bacteria by macrophages (13). Furthermore, a study on basal metazoan coral (*Euphyllia ancora*) revealed that all three domains in Vg not only interact with bacteria but also enhance phagocytosis (26). In the present study, we have shown that both DUF1943 and VWD domains bind with different bacterial strains *via* the signature components of LTA and LPS, which were similar with the results in zebrafish (13), suggesting the bacteria-binding function of these domains may be conserved across vertebrates and invertebrates. Interestingly, only VWD domain exhibited strong bacteria growth inhibition activity. Although we did not test the antibacterial function of VWD in Vg of different species, the conserved amino acid residues mutated protein showed significantly lower bacteria growth inhibition activity, which indicated that antibacterial activity may act as a common signature for VWD from different species. Moreover, coimmunoprecipitation assay showed that only the DUF1943 domain binds with *Es*pIgR, a crucial phagocytic receptor in arthropods, and strongly promotes hemocyte phagocytosis. However, it is still unclear whether the other two domains also regulate phagocytosis by different cell membrane receptors.

As a type I transmembrane glycoprotein, pIgR in different species shares four similar components: an intracellular region, a transmembrane region, a cleavage region, and an extracellular ligand-binding region (27). The Ig domains are located in the extracellular region (secretory component (SC)). Therefore, the N-terminal ligand-binding domain plays central roles in binding polymeric immunoglobulins. PIgR acts as a receptor for pIg and transports pIgA/pIgM across intestinal epithelial cells in vertebrates (28). In addition, pIgR and SC-mediated protection prevent the invasion of pathogenic microorganisms at mucosal surfaces (29, 30). Several studies on pIgR found that certain microorganisms, such as *Streptococcus pneumoniae*, hijack pIgR to their own benefit during the invasion of host cells, indicating the crucial role of pIgR on endocytosis (31, 32). Recent studies on shrimp have indicated that pIgR is a WSSV receptor and that WSSV enters shrimp cells *via* the pIgR-CaM-Clathrin endocytosis pathway (22). Our results demonstrated that *Es*pIgR is a phagocytic receptor for bacterial binding to *Es*Vg, which highlights the important function of pIgR in crustacea and also indicates the multiple functions of pIgR in innate immunity.

In summary, three conserved domains, LPD_N, DUF1943, and VWD, were identified in crab Vg and found to facilitate diverse immunological functions. The DUF1943 and VWD domains bind with invading bacteria, and the VWD domain inhibits extracellular bacterial proliferation. Furthermore, the DUF1943 domain interacts with *Es*pIgR at the cell membrane to promote hemocyte phagocytosis, thus ultimately fostering innate immunity in crabs (Fig. 9*E*).

## Experimental procedures

### Animals and primary-cultured hemocytes

Our experiments followed the protocol approved by the East China Normal University Animal Care and Use Committee (protocol license number AR2012/12017) in direct accordance with the Ministry of Science and Technology (China) for animal care guidelines. Healthy nonantibiotic or antifungal-fed *E. sinensis* crabs (100 ± 10 g each for adults and 20 ± 2 g each for naïve crabs) were obtained from the Songjiang aquatic farm (Shanghai, China). After quickly transferring them to the East China Normal University, all crabs were maintained in filtered and aerated freshwater provided with plenty of oxygen and fed daily with a commercial formulated diet containing no antibiotics.

Primary cultures of *E. sinensis* hemocytes were generated following established techniques (33). From the adult crabs, isolated hemocytes were gently resuspended in Leibovitz’s L-15 medium (Sigma-Aldrich, Santa Clara, USA) supplemented with 1% antibiotics (10,000 U/ml penicillin, 10,000 μg/ml streptomycin [Gibco, Waltham, USA]) and 0.2 mM NaCl (676 ± 5.22 mOsm/kg), at pH 7.20 to 7.40. Hemocytes were then counted with an automated cell counter (Invitrogen Countess, Waltham, USA) before seeding 4 ml (1 × 10^6^ cells/ml) each into 60-mm dishes.

### Immune stimulation and sample collection

*S. aureus* and *V. parahaemolyticus* were cultured, collected, and resuspended in sterile PBS (137 mM NaCl, 2.7 mM KCl, 10 mM Na_2_HPO_4_, 2 mM KH_2_PO_4_ [pH 7.4]). Bacterial counts were determined by plating the diluted suspension onto agar plates.

For the *in vitro* bacterial stimulation, cultured *S. aureus* or *V. parahaemolyticus* (1 × 10^7^ microbes per dish, 50 μl) was added separately to the hemocyte-cultured dish, and sterile PBS (50 μl) was used as the control. Total RNA was collected from hemocytes at specific times after stimulation. Three or more crabs were used per sample. First-strand cDNA was synthesized using a Reverse Transcriptase Kit (Takara, Osaka, Japan) following the manufacturer’s instructions.

For the *in vivo* bacterial infection, *S. aureus* or *V. parahaemolyticus* (1 × 10^8^ CFU per crab, 200 μl) was injected into the hemolymph from the nonsclerotized membrane of the posterior walking leg, with sterile PBS (200 μl) served as control. Total RNA was collected from hemocytes at specific time points after infection. At least three crabs were used per sample.

### Genomic sequencing, assembly, annotation, and phylogenetic analysis

A fragment containing a full ORF encoding a protein with pIgR was identified from NCBI, and this was designated as *Es*pIgR. Its full-length cDNA was amplified by a pair of gene-specific primers and then resequenced to confirm the accuracy. A similarity analysis was carried out using Clustalx from EMBL, with signal peptides and domain architecture predicted respectively by the SignalP v4.1 Server (http://www.cbs.dtu.dk/services/SignalP/) and SMART (http://smart.embl-heidelberg.de/), and protein 3D structure was predicted by the Swiss-PdbViewer (https://spdbv.vital-it.ch/). A phylogenic tree was constructed based on the deduced amino acid sequences of *Es*pIgR and other known pIgR by the neighbor-joining (NJ) algorithm using MEGA v6.0 software, bootstrap trials were replicated 1000 times.

### RNA interference assay

cDNA fragments of *Es*pIgR were PCR-amplified using primers linked to the T7 promoter (Table S1), to then serve as templates for producing siRNA with an *in vitro* T7 Transcription Kit (Fermentas, Burlington, Canada), with GFP siRNA purchased from the GenePharma Company (Shanghai, China) as the control. For the *in vitro* RNAi, the siRNA was dissolved in RNase-free water and transfected into *E. sinensis* primary-cultured hemocytes by using Lipofectamine 3000 (Thermo Fisher, Waltham, USA) to a final concentration of 10-nM. For the *in vivo* RNAi, the siRNA (1 μg/g) was injected into the crab hemolymph. To extend the RNAi effect for crab survival assay, a second injection was administered to each crab at 2 days after the first-round injection. To determine RNAi efficiency, real-time RT-PCR was used with siGFP RNA as the control. After confirming the efficiency of RNAi assay, *S. aureus* or *V. parahaemolyticus* was used to stimulate gene-silenced hemocytes *in vitro* or injected into gene-silenced crabs *in vivo*.

### Gene expression profile analysis

Expression levels of *Es*pIgR in the differently treated hemocytes were determined with quantitative RT-PCR (qRT-PCR; primers shown in Table S1) using a QuantStudio 5 Real-Time System (Applied Biosystems, USA) and SYBR Premix Ex Taq (Tli RNaseH Plus; TaKaRa, Osaka, Japan). Reaction conditions were used as following: 94 °C for 3 min, then 40 cycles at 94 °C for 10 s and 60 °C for 1 min, followed by melting from 65 to 95 °C. The gene expression levels were derived by the 2^−ΔΔCT^ calculation method and normalized to the control group. Three independent experiments were performed and the results are presented as mean ± SD

### Expression and purification of recombinant protein

The recombinant expression plasmids pET-28a-LPD_N, pET-28a-DUF1943, and pET-28a-VWD were transformed into *E. coli* Transetta (DE3) (TransGen, Beijing, China). Single colonies of transformants were chosen and cultured to the mid-log phase (OD600 = 0.6). For expression of recombinant protein, four conditions were tested: 0.25 mM isopropyl b-D-1-thiogalactopyranoside (IPTG) for 3 h at 30 or 37 °C, and 1 mM IPTG for 3 h at 30 or 37 °C. The lysate of the induced cells was then collected after sonication and analyzed by 12% SDS-PAGE. Subsequently, the lysates were resuspended in binding buffer and the desired fusion protein purified using a Ni-NTA HisTrap FF crude column and washed with imidazole elution buffer (50 mM Tris-HCl, 300 mM NaCl, PH 7.5 and 250 mM imidazole) at 1 ml/min.

### Assessment of crab survival and bacterial clearance assay

Crabs were randomly divided into eight groups each with 30 animals. After pretreating the crab with recombinant DUF1943 protein (rDUF1943), rDUF1943 plus *Es*pIgR siRNA, or GFP siRNA, all crabs were injected with *S. aureus* or *V. parahaemolyticus* (1 × 10^9^ CFU per crab, 200 μl). Dead crabs in each group were counted daily, and survival was tabulated over 5 days. Three days after the bacterial injections, each crab’s hemolymph was collected, diluted, and cultured overnight on solid LB plates, then bacterial colonies per plate were counted.

### Microorganism-binding activity assay

Gram-positive bacteria (*S. aureus* and *Bacillus subtilis*) and Gram-negative bacteria (*Aeromonas hydrophila*, *V. parahaemolyticus*, *Vibrio anguillarum*, and *Edwardsiella tarda*) were cultured overnight in Luria–Bertani (LB) medium. The microorganisms were collected by centrifugation at 6000*g* and resuspended in 2 ml 1× TBS (Beyotime, China). Then, the microorganisms (2 × 10^7^ CFU/ml in 500 ml of TBS) were incubated with 150 mg different purified Vg domain proteins for 1 h at 37 °C. After 6000*g* centrifugation for 5 min, the microorganisms were collected once again, washed three times with TBS, and finally resuspended in TBS. SDS-PAGE loading buffer was added to each sample and then boiled for 8 min. The microorganism-binding activity of different *Es*Vg domains was measured by western blotting with antiHis-tag mouse antibody (Beyotime, China).

### LTA and LPS binding activity assay

Assay for binding of rDUF1943 and rVWD to LTA and LPS was conducted as previously described (13). Briefly, 100 μl LTA (100 μg/ml), LPS (100 μg/ml), or PBS (pH 7.4) was mixed with 10 μg of rDUF1943 or rVWD, respectively, and incubated for 1 h at 25 °C. After that, *S. aureus* cells (1 × 10^7^ cells) were introduced in the LTA and PBS groups, and *V. parahaemolyticus* cells (1 × 10^7^ cells) in the LPS and PBS groups. Bacterial cells were collected by incubation and centrifugation and the bacterial pellets were resuspended in 100 μl of PBS. Subsequently, 10 μl of each bacterial sample was electrophoresed on a 12% SDS-PAGE gel and immunostained.

To quantify the binding of rDUF1943 and rVWD to LTA and LPS, ELISA was performed as described before (13). Briefly, the recombinant proteins were individually diluted in water to a concentration of 1 μM. Fifty microliter of rDUF1943, rVWD, or TRX-His-tag peptide was applied to each well of a 96-well microplate and air-dried overnight at 20 °C. The plates were incubated at 60 °C for 30 min and blocked with 100 μl of 1 mg/ml BSA in PBS at 37 °C for 2 h. After washing four times with PBST, biotin-labeled LTA or LPS was added into each well and incubated at 25 °C for 3 h, washed five times with 200 μl of PBST, and incubated with 100 μl of streptavidin–HRP (TransGen Biotech, Beijing, China) with 0.1 mg/ml BSA in PBS for 1 h. The wells were then washed and added with 75 μl of 0.4 mg/ml O-phenylenedi-amine (Amresco) in the buffer and reacted at 37 °C for 10 min. Subsequently, H_2_SO_4_ (25 μl of 2 M) was added into each well to terminate the reaction, and OD492 was monitored by a microplate reader (Bio-Rad, USA).

### Antimicrobial activity assays

Growth curves of *S. aureus*, *V. parahaemolyticus*, *M. luteus*, and *E. coli* that cultured with recombinant LPD_N, DUF1943, VWD, or mutated VWD protein were detected according to the method that described previously (34). Site-directed mutagenesis was performed with Quik-Change kit (Stratagene) using primers listed in Table S1, and the mutation efficiency was confirmed by sequencing three individual clones from plasmids. The bacteria were cultured in the broth (LB and YPD, respectively) overnight until the OD600 reached about 0.8 to 1.0. After overnight culture, the purified recombinant protein was added to a final concentration of 20 mg/ml or 150 mg/ml, with Tris-buffered saline (TBS) as a control. The samples were incubated at 37 °C and centrifuged at 180 rpm, then the OD600 was measured every 2 h.

### Coimmunoprecipitation

To confirm the interaction between *Es*Vg and *Es*pIgR *in vivo*, HEK293T cells were cultured for coimmunoprecipitation assays following previously reported methods (35). Briefly, HEK293T cells were first seeded in 24-well plates and then cultured overnight in 37 °C DMEM (Life Technologies/Invitrogen, Carlsbad, CA) supplemented with 10% FBS (HyClone, Logan, UT) and 1% antibiotic-antimycotic solution (Life Technologies). After 24 h, cells were cotransfected with different Flag-tagged *Es*Vg domain plasmids (pcDNA3.0-*Es*Vg-LPD_N, pcDNA3.0-*Es*Vg-DUF1943, pcDNA3.0-*Es*Vg-VWD, and pcDNA3.0-*Es*Vg-△DUF1943) and the HA-tagged extracellular domain of *Es*pIgR (pcDNA3.0-EspIgR-extracellular domain), respectively, using Lipofectamine 3000 transfection reagent (Thermo Fisher, Waltham, USA) according to the manufacturer’s instructions. At 36 h posttransfection, the HEK293T cells were washed twice with PBS before their proteins were extracted with an RIPA buffer, followed by centrifugation at 14,000*g* for 10 min. The supernatant was then precleared with 30 μl of Protein A beads for 40 min at 4 °C with shaking and centrifuged at 12,000*g* for 10 min to remove the beads. The ensuing supernatant was incubated with 10 μg of Flag and HA antibodies overnight at 4 °C under gentle rotation, added with Protein A beads, and incubated at 4 °C for 1 h to capture the antibodies. The centrifugation and PBS washing were employed to collect the beads, then resuspended in the SDS-PAGE loading buffer for separation by SDS-PAGE and western blot assays (using the Flag or HA antibody). All images were collected using an Odyssey CLx (LI-COR, Lincoln, USA).

### Fluorescent labeling of bacteria and phagocytosis assay

Overnight-cultured *S. aureus* and *V. parahaemolyticus* were heat-killed and fluorescein isothiocyanate (Sigma, USA) conjugated. The bacterial suspension (1 × 10^8^ microbes per milliliter, 1 ml) in PBS was mixed and incubated with 500 μg of recombinant proteins by gentle rotation for 1 h at 28 °C to ensure full coating. The bacteria were pelleted and washed three times with PBS by centrifugation.

For the *in vitro* analysis, overnight cultures of bacteria were heat-inactivated at 72 °C for 20 min and collected by centrifugation, then cultured hemocytes (1 × 10^6^ cells) were stimulated first with heat-killed bacteria (1 × 10^7^ CFU) for 12 h and washed with PBS twice before the addition of approximately 1 × 10^6^ (40 μl) FITC-conjugated microbes coated with recombinant proteins. They were then incubated for 40 min at room temperature. For the *in vivo* analysis, the bacterial suspension (100 μl) was injected into the hemolymph of crab at one of the posterior walking legs. One hour later, the hemocytes (6 × 10^5^ cells) were isolated from the other of the posterior walking legs and centrifuged at 300*g* for 10 min at 4 °C and washed with PBS three times. After that, the phagocytosis rate in 1 ml of each sample was determined by flow cytometry using a CytoFLEX apparatus (Beckman, USA), and the data were analyzed using CytExpert software. At least 10,000 hemocytes taken from three crabs were counted for each sample. The experiments were repeated three times. Three days after the bacterial injections, each crab’s hemolymph or medium supernatant was drawn, diluted, and cultured overnight on solid LB plates, then bacterial colonies per plate were counted.

### Chlorpromazine injection

To detect the endocytosis of bacteria, the clathrin-dependent endocytosis inhibitor chlorpromazine (CPZ, Sangon Biotech, Shanghai, China) and 500 μg of recombinant proteins fully coated with bacteria (1 × 10^8^ CFU) were added to primary-cultured crab hemocytes medium according to previous described method (22). The phagocytosis rate in 1 ml of each sample was determined by flow cytometry using a CytoFLEX apparatus (Beckman, USA), and the data were analyzed using CytExpert software. The resulting plots are representative of three independent assays, and the phagocytosis ratios were calculated from those three tests.

## Data availability

All data are contained within the article.

## Conflict of interest

The authors declare that they have no conflicts of interest with the contents of this article.
