# Supplementary material for: Distinct vitellogenin domains differentially regulate immunological outcomes in invertebrates
Source: J Biol Chem. 2020 Dec 1;296:100060. doi: 10.1074/jbc.RA120.015686 (PMC7949091; doi:10.1074/jbc.RA120.015686)
Supplement: Table S1 [file mmc1.docx]

**Tab.S1 Sequences of primers used for analysis**

| **Primers** | **Sequence (5’-3’)** |
| --- | --- |
| **cDNA cloning** |  |
| *Es*pIgR-F | ATGTGCAAGTGTTCCTTTGTGC |
| *Es*pIgR-R | CTATTCTTTTTTCTCGTCTTCCCG |
| *Es*Vg-F | GATGCCGTTCCTCGTTGC |
| *Es*Vg-R | AATGTTTGTCAGCAGGGAGC |
| LPD_N-F | ATGACCAACCGCACAGCC |
| LPD_N-R | GCCGGGAGCGTAGATAAG |
| DUF1943-F | GGGTTTATCCTGAGCCATTT |
| DUF1943-R | CGAGGTCGTAGCACATCTTT |
| VWD-F | CCTACTATGCGTGGATGCC' |
| VWD-R | CTCGTGGAGGTTTGTGGC |
| **Quantitative real-time PCR** |  |
| Q-*Es*Vg-F | CACCTCCGCCTATGATGAC |
| Q-*Es*Vg-F | TTCGGGTAGACCTTGATGC |
| Q-*Es*pIgR-F | GACGTGTACGGTAACGTGTATG |
| Q-*Es*pIgR-R | GGTTTGTCGGTTTGATTGTG |
| Q-Actin-F | GCATCCACGAGACCACTTACA |
| Q-Actin-R | CTCCTGCTTGCTGATCCACATC |
| **Plasmid construction** |  |
| *Es*pIgR-pET28a-His-F | CCGGAATTCAACCAGATAGTCGTGAACCCC *EcoRI* |
| *Es*pIgR-pET28a-His-R | CCCAAGCTTCTAGACTCTGTACCGAGTTTCACCAA *HindIII* |
| LPD_N-pET28a-His-F | CCGGAATTCTTCATTCCGGGCAAGACCTAT *EcoRI* |
| LPD_N-pET28a-His-R | CCCAAGCTTCTACAAATGGCTCAGGATAAACCC *HindIII* |
| DUF1943-pET28a-His-F | CCGGAATTCAGGAAGTACTCCCGCAACATT *EcoRI* |
| DUF1943-pET28a-His-R | CCCAAGCTTCTATAGGAACGCCTCACTTCGTA *HindIII* |
| VWD-pET28a-His-F | CCGGAATTCCTGTCCAACTCCGTTCCAC *EcoRI* |
| VWD-pET28a-His-R | CCCAAGCTTCTAGTCGCTCCTGAGTCTGTCGT *HindIII* |
| *Es*pIgR-PcDNA3.0-HA-F | CCGGAATTCTGAACCAGATAGTCGTGAACCCC *EcoRI* |
| *Es*pIgR-PcDNA3.0-HA-R | ATAAGAATGCGGCCGCCTAGACTCTGTACCGAGTTTCACCAA *NotI* |
| LPD_N- PcDNA3.0-Flag-F | CCGGAATTCTGTTCATTCCGGGCAAGACCTAT *EcoRI* |
| LPD_N- PcDNA3.0-Flag-R | ATAAGAATGCGGCCGCCTACAAATGGCTCAGGATAAACCC *NotI* |
| DUF1943- PcDNA3.0-Flag-F | CCGGAATTCTGAGGAAGTACTCCCGCAACATT *EcoRI* |
| DUF1943- PcDNA3.0-Flag-R | ATAAGAATGCGGCCGCCTATAGGAACGCCTCACTTCGTA *NotI* |
| VWD- PcDNA3.0-Flag-F | CCGGAATTCTGCTGTCCAACTCCGTTCCAC *EcoRI* |
| VWD- PcDNA3.0-Flag-R | ATAAGAATGCGGCCGCCTAGTCGCTCCTGAGTCTGTCGT *NotI* |
| **Site-directed mutagenesis**  VWD-T20A-F  VWD-T20A-R  VWD-F21A-F  VWD-F21A-R  VWD-V35A-F  VWD-V35A-R  VWD-L36A-F  VWD-L36A-R  **RNA interference** | TCCTCGCGTTCGACGGCGCTCTTC  AGGAGCTCCGTGTCAGCCAC  TCACCGCGGACGGCGCTCTTCTTC  GTGAGGAGCTCCGTGTCAGC  ACGCTGCAAGGCGCTGCTCTCCTC  CCTTGCAGCGTGAGCGAGGC  AGGTTGCGCTCTCCTCTGTGCC  AACCTTGCAGCGTGAGCGAG |
| siRNA-*Es*pIgR-F1 | GCUCAACAAUACGGCGAUUTT |
| siRNA-*Es*pIgR-R1 | AAUCGCCGUAUUGUUGAGCTT |
| siRNA-*Es*pIgR-F2 | GCGGACGAGAAGACAGAAUTT |
| siRNA-*Es*pIgR-R2 | AUUCUGUCUUCUCGUCCGCTT |
| siRNA-GFP-F | UAAUACGACUCACUAUAGGG |
| siRNA-GFP-R | CCCUAUAGUGAGUCGUAUUA |
